# Platelet aggregometry assay for evaluating the effects of platelet agonists and antiplatelet compounds on platelet function *in vitro*

**DOI:** 10.1016/j.mex.2018.12.012

**Published:** 2018-12-26

**Authors:** Alexandros Tsoupras, Ioannis Zabetakis, Ronan Lordan

**Affiliations:** Department of Biological Sciences, University of Limerick, Limerick, V94 T9PX, Ireland

**Keywords:** Human platelet aggregometry assay, ADP, adenosine diphosphate, LTA, light transmission aggregometry, PAF, platelet-activating factor, PPP, platelet-poor plasma, PRP, platelet-rich plasma, Platelet aggregation, Light transmission aggregometry, Platelet-activating factor, Thrombin, Antiplatelet, Agonist, *In vitro*, Inhibitors

## Abstract

Platelet aggregometry assays are generally used for the analysis of platelet function but can also be adapted for further research and therapy focused applications. This method describes the procedures for the preparation of human platelet-rich plasma (PRP) and platelet-poor plasma (PPP) for the assessment of human platelet aggregation induced by agonists such as platelet-activating factor (PAF), thrombin, collagen, adenosine diphosphate (ADP), arachidonic acid, *etc.*

• This method can be applied *in vitro* to evaluate the aggregatory effects of these agonists and to assess the antiaggregatory effects of several bioactive antiplatelet agents (compounds of natural or pharmacological origin) in human PRP.
• This versatile method can be used in both basic and clinical research for the assessment of platelet aggregation (a major cardiovascular risk factor), platelet agonists, and inhibitors, in physiological or pathological conditions.
• This method can be adapted to assess platelet activity in postprandial and intervention studies *ex vivo*.

## Graphical abstract

**Figure:**
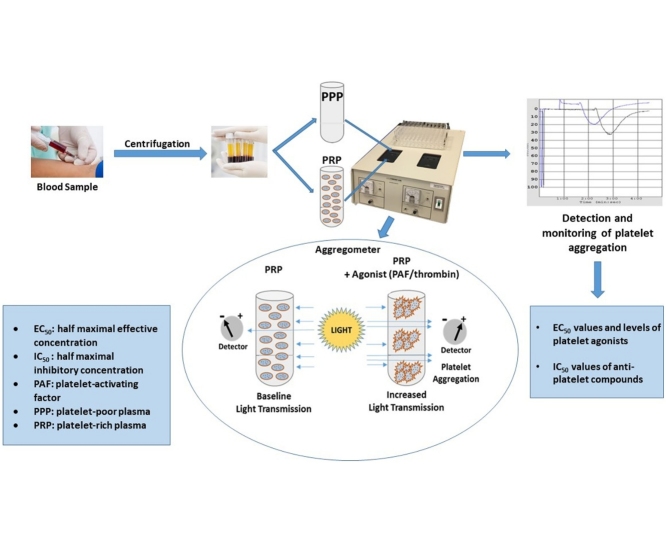

**Specifications Table**

**Table:** 

**Subject Area**	Agricultural and Biological Sciences Medicine and Dentistry
**More specific subject area**:	Haematology
**Method name**:	Human platelet aggregometry assay
**Name and reference of original method**	Tsoupras, A., Lordan, R., Demuru, M., Shiels, K., Saha, S. K., Nasopoulou, C. and Zabetakis, I. (2018) 'Structural elucidation of Irish organic farmed salmon (*Salmo salar*) polar lipids with antithrombotic activities', *Marine Drugs*, 16(6), 176.
**Resource availability**	All reagents and instruments indicated are commercially available. Sources of critical components are clearly indicated in the manuscript.

## Background

Human platelets are crucially involved in both normal haemostasis, pathological bleeding, and thrombosis. These platelets contribute greatly to vessel constriction and repair, host defence, and when acting together with other cells (*i.e.* white blood cells, endothelial, or smooth muscle cells) they also participate in inflammatory pathologies, such as the promotion of atherosclerosis, tumor growth, and metastasis [[2], [3], [4], [5], [6]]. During the interaction between platelets and the vessel wall, at the site of damage platelets are rapidly engaged in a sequence of functional responses including various steps in platelet activation, adhesion, spreading, shape change, aggregation, release reaction, exposure of a procoagulant surface, and clot retraction. These processes lead to the formation of a haemostatic plug that occludes the site of injury to prevent blood loss. An increased risk of bleeding could be present when the platelet count is reduced and/or one of their functions is defective. Conversely, improper thrombus formation could be due to a growth in platelet count or reactivity. Activated platelets adhere and aggregate within atherosclerotic lesions, forming occluding arterial thrombi that may result in atherothrombotic manifestations such as stroke or myocardial infarction, two of the major causes of morbidity and mortality in the Western world [2].

Several agonists of platelet activation and aggregation are implicated in all steps of these pathologies, such as the platelet-activating factor (PAF) and thrombin. Antiplatelet compounds of either natural, dietary or pharmacological origin have been found to beneficially reduce these effects [2]. Thus, the European Food Safety Authority (EFSA) has accepted that decreasing platelet aggregation in subjects with platelet activation during sustained exposure to a food or food constituent over four weeks is a beneficial physiological effect [7]. A food constituent that demonstrates these properties can be granted a valid health claim from EFSA [8]. Therefore, reliable platelet functions tests are required to assess such a wide range of functions, effects of platelet aggregatory agonists, and antiplatelet compounds. The revolutionary platelet aggregation test in platelet-rich plasma (PRP), called light transmission aggregometry (LTA), was developed by Born [9] and O’Brien [10] in the 1960s. LTA is considered the gold standard for testing platelet function and it is still in use for evaluating and monitoring platelet defects and the effects of platelet aggregation agonists and antiplatelet compounds.

**Fig. 1:**
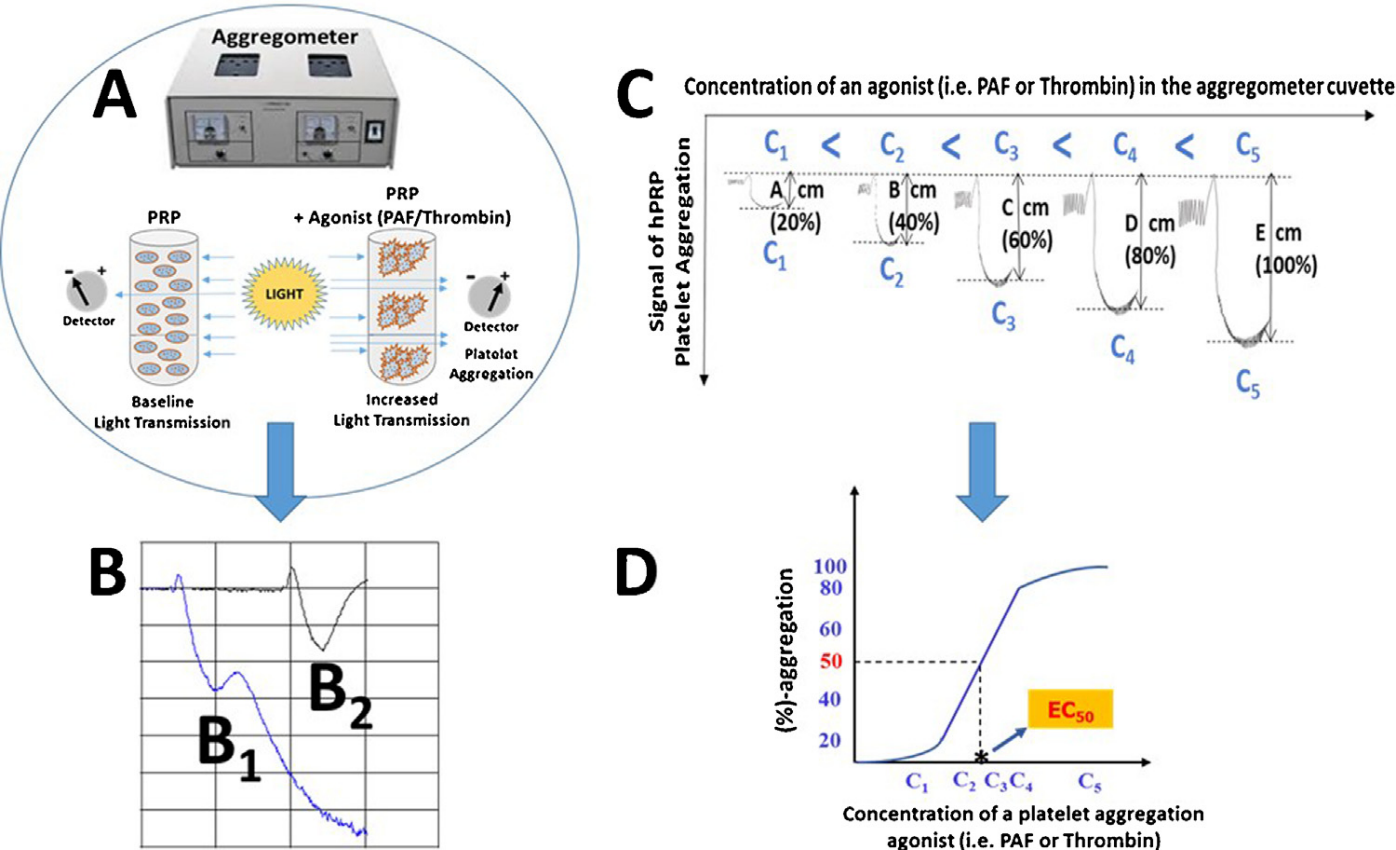
Application of LTA assay for evaluating and monitoring the effect of several agonists (*i.e.* PAF or thrombin) on platelet aggregation of PRP. (A): Detection of increased light transmission during platelet aggregation; baseline light transmission is acquired when platelets of PRP suspensions are in an aggregometer cuvette in the absence of an agonist. Increased light transmission is detected when platelets start to aggregate in the presence of an agonist in the aggregometer cuvette leading to the development of an aggregation curve. (B): Characteristic examples of an aggregation curve of a maximum irreversible platelet aggregation [B_1_: Trace 1 (blue)] and of an aggregation curve of a maximum-reversible platelet aggregation [B_2_: trace 2 (black)]. The latent aggregation curve (B_2_) of maximum-reversible platelet aggregation of hPRP induced by an agonist (PAF or thrombin) is determined as the 100% aggregation of platelets. (C): The addition of several concentrations of an agonist (PAF or thrombin) within a specific range can induce reversible platelet aggregation in a concentration dependent manner; *i.e.* the higher the concentration of the agonist (C5 > C4 > C3 > C2 > C1) the higher the platelet aggregation curve (E > D > C > B > A) that is obtained by the aggregometer detector of the LTA assay. (D): A linear relationship exists between the concentrations of an agonist (*i.e.* PAF or thrombin) within a specific range that induces platelet aggregation within the 20%–80% of the maximum-reversible platelet aggregation of hPRP. From this linear curve the concentration of the agonist needed to induce 50% of platelet aggregation can be calculated, which is known as the EC_50_ value of the specific agonist for platelet aggregation. The lower the EC_50_ value for an agonist the higher the potency of its platelet aggregation effect. All experiments must be performed in triplicate (n = 3), using a different donor’s blood sample for each replicate, to ensure reproducibility. **Abbreviations:** LTA: light transmission aggregometry; PAF: platelet-activating factor; hPRP: human platelet-rich plasma.

LTA measures the transmission of light through a sample of platelets in suspension (PRP, washed platelets or gel-filtered platelets), which increases when platelets are aggregated by an agonist such as PAF, thrombin, ADP, arachidonic acid (AA), collagen, epinephrine (EPI), *etc.* (Fig. 1A). LTA is a time-consuming and technically challenging technique that is affected by many preanalytical and analytical variables, thus it must be carefully controlled and performed by expert personnel in specialised laboratories [11].

The method outlined in this manuscript describes the processing required to prepare human PRP and PPP and the analytical procedures required to assess platelet aggregation of PRP induced by agonists such as PAF and thrombin using LTA *in vitro*. Previous research relating to antiplatelet compounds or antithrombotic food constituents focused on the use of washed rabbit platelets or rabbit PRP [[12], [13], [14], [15], [16], [17], [18], [19]]. This method describes the assessment of these antiplatelet compounds using human PRP *in vitro* [1]. This is important for preclinical evaluation of the health benefits or side-effects of these antiplatelet compounds on platelet function and cardiovascular health before the use of these compounds in *in vivo*, ex *vivo,* or clinical research.

## Materials, methods and applications

### Materials and reagents

All plastic consumables, reagents, and solvents were of analytical grade and were purchased from Fisher Scientific Ltd. (Dublin, Ireland). 20 gauge (G) safety needles and evacuated sodium citrate S-monovettes® for blood sampling were purchased from Sarstedt Ltd. (Wexford, Ireland). Bioassays on human PRP (hPRP) were carried out on a Chrono-log-490 two channel turbidimetric platelet aggregometer (Havertown, PA, USA), coupled to the accompanying AGGRO/LINK software package. All platelet aggregation consumables were purchased from Labmedics LLP (Abingdon on Thames, UK). Standard PAF, thrombin and bovine serum albumin (BSA) were purchased from Sigma Aldrich (Wicklow, Ireland). Centrifugations were carried out on an Eppendorf 5702R centrifuge (Eppendorf Ltd., Stevenage, UK). Spectrophotometric analysis was carried out on a Shimadzu UV-1800 spectrophotometer (Kyoto, Japan).

### Preparation of PAF

PAF is purchased as a crystalline substance (Sigma Aldrich, Wicklow, Ireland). Dilute the PAF in an appropriate amount of a chloroform/methanol (1:1 v/v) solution so that the stock concentration is 10^−4^ M, which should then be aliquoted for storage at −20 °C. When ready for use, the aliquoted stock solutions should be evaporated under a stream of nitrogen and re-dissolved in BSA-saline (2.5 mg BSA/mL 0.9% saline). This should then be serially diluted in BSA-saline to obtain solutions of PAF dissolved in BSA-saline with a final concentration in the cuvette ranging from 2.6 × 10^−8^ to 2.6 × 10^−5^ mol/L.

### Preparation of thrombin

Crystalline standard active thrombin (Sigma Aldrich, Wicklow, Ireland) should be dissolved in saline, so that the stock concentration is 0.1 U/μL and this solution can be aliquoted for storage at −80 °C. When ready for use, the aliquoted stock solutions should be diluted in appropriate amounts of saline to obtain solutions of thrombin with a final concentration in the aggregometer cuvette ranging from 0.01mU/μL to 1mU/μL.

### Preparation of platelet-rich plasma (PRP) and platelet-poor plasma (PPP)

Blood withdrawal and the preparation and processing of human PRP can be performed as previously described [1] and outlined in detail below:

1 For PRP isolation, volunteers donate fasting blood samples (for at least 8 h).
2 Blood should collected from the median cubital vein or cephalic vein of healthy volunteers *via* venepuncture using a 20 G safety needle. Blood must be drawn into sodium citrate anticoagulant S-monovettes (0.106 mol/L in a 1:10 ratio of citrate to blood; Sarstedt Ltd., Wexford, Ireland) using the aspiration method (or similar collection systems and venepuncture methods). The monovette must be gently inverted three times to ensure effective mixing of the anticoagulant.
3 The collected blood samples should then centrifuged at 194 x g for 18 min at 24 °C with no brake applied to prevent any platelet activation. The supernatant PRP must then gently transferred to polypropylene tubes using a polypropylene Pasteur pipette and the PRP is stored at 24 °C.
4 The PPP is obtained by further centrifuging the remainder of the PRP specimen in the monovette at 1465 x g for 20 min at 24 °C, again with no brake applied.
5 PRP can be adjusted to 500,000 platelets/μL if required by addition of a respective volume of PPP according to the absorbance of the PRP measured. The absorbance is measured using a spectrophotometer set at 530 nm and the PPP is used as a blank. 1 mL of PPP and PRP are placed in plastic cuvettes and the absorbance is measured. An absorbance of 0.8 is equivalent to 500,000 platelets/μL.
6 All procedures should take place at 24 °C and should be carried out within 2.5 h of the initial blood draw for optimum performance.

#### Notes on the preparation of human platelet-rich plasma

• Ensure to use a wide bore needle for blood withdrawal if the one recommended in this protocol is not available to prevent unnecessary platelet aggregation or activation.
• Ensure that the monovettes or similar collection tubes are in date before use.
• Venepuncture should be clean without the requirement of probing for a vein, to avoid platelet activation and haemolysis. Follow the local standard operating protocols for venepuncture.
• It is recommended that platelets are handled and stored in polypropylene, polyethylene or polycarbonate labware. In addition, stored platelets should always be sealed with a cap to ensure that the pH of the hPRP remains stable. Note that glass containers, vigorous movement of containers or pipettes can cause platelet activation. Deviation of these recommendations may lead to a loss of platelet function or premature platelet activation.
• Blood and intact platelets should preferentially be handled at room temperature (approximately 24 °C). Note that platelets at 4 °C can activate and suffer loss of function.
• Many pharmaceutical compounds and various reagents are known to interfere with platelet function. Ensure that the potential donors have not used the following drugs two weeks prior to blood draw: anti-histamines, antibiotics, aspirin, anaesthetics, dextran, PAF-inhibitors such as Rupatadine or some herbal medicines, non-steroidal anti-inflammatory medication (Aspirin, Clopidogrel, Cilostazol, Ibuprofen, Ticlopidine, Ticagrelor, Prasugrel – or any other vascular or cardiovascular related medications).
• Some foods and beverages such as coffee, garlic, and wine can also impact platelet function. Ensure that volunteers have been fasting for a minimum of 8 h prior to testing.
• The preparation of thrombin and PAF is described in this methodology, however, other agonists can just as easily be applied to these methods.

### Aggregometry protocols

#### Basic aggregometry protocol

The aim of this protocol is to describe basic aggregometry testing. However, consult the manufacturer’s guidelines associated with the particular model of aggregometer and adjust this method accordingly. The basic aggregometry protocol in human PRP can be performed as previously described [1] with modification as outlined below:

1 Draw and process the blood as described in section 2.4.
2 Prior to testing, add 250 μL of PPP to a glass aggregometer cuvette with a spacer attached (see note below) without a stirrer and place it in the appropriate well in the aggregometer.
3 Then pipette 250 μL of hPRP into another glass cuvette, which is placed in a Chrono-log-490 two channel turbidimetric platelet aggregometer (Havertown, PA, USA), coupled to the accompanying AGGRO/LINK software package. This is a Born type aggregometer with a fixed wave spectrophotometer and a sample chamber where the cuvette filled with PRP is placed at 37 °C. Add a small disposable stirrer to the cuvette spinning at 1200 rpm and allow the cuvette to prewarm before testing. This allows assay to mimic the *in vivo* conditions of blood flow.
4 Then, automatically calibrate (zero) the PRP against the PPP (follow the manufacturer’s instructions). In doing so, the instrument is set to 100% transmission and then 0% transmission in order to gauge the possible parameters. When complete, wait 30 s to establish a stable baseline, prior to the addition of any agonists.
5 Baseline light transmission is obtained when platelets of hPRP suspensions are in an aggregometer cuvette in the absence of an agonist, while increased light transmission is detected when platelets start to aggregate in the presence of an agonist in the aggregometer cuvette (Fig. 1A). According to the detection of these changes of the light transmission specific aggregation curves are acquired. A characteristic example of an aggregation curve of a maximum-irreversible platelet aggregation is shown in Fig. 1(B_1_), while a characteristic example of an aggregation curve of a maximum-reversible platelet aggregation is shown in Fig. 1(B_2_).
6 The latent aggregation curve (Fig. 1B_2_) of maximum-reversible platelet aggregation of hPRP induced by an agonist (PAF or thrombin) is determined as the 100% aggregation of platelets. This aggregation curve is very important to determine in each PRP sample in order to carry out some of the applications of LTA, including the evaluation of the effects of platelet agonists and antiplatelet compounds.

**Notes on the Aggregometry Protocol**:

• Use pipette tips with a wide orifice to prevent damage or activation of the platelets.
• Ensure that the PRP is pipetted directly into the centre of the cuvette to prevent activation of the platelets by the glass cuvettes.
• Avoid unnecessary pumping or vigorous pipetting techniques to prevent activation of the platelets or bubble formation.
• Wear gloves when handling clinical material for personal protection, but also to prevent any smudges or fingerprints that may affect the aggregometer analysing the contents of the glass cuvettes. Clean and inspect all cuvettes before use with a lint free disposable tissue.
• Dispose of all clinical waste and sharps correctly according following local standard operating procedures.
• The Chrono-log cuvettes can be fitted with a spacer. This is a small rubber object placed underneath the cuvette to raise the height of the cuvette. Ordinarily, 500 μL of PRP would be required to test in the Chrono-log aggregometer. However, spacers allow for a lower volume of PRP to be used.
• Some aggregometer stirrers are set at 1000 RPM as opposed to 1200 RPM, which is satisfactory.

#### Application of LTA for the evaluation of platelet agonists on platelet aggregation of PRP (EC_50_ values)

The application of LTA for evaluating the effects of agonists (*i.e.* PAF or thrombin) on platelet aggregation of PRP can be achieved as previously described [20], with some modifications. Briefly:

1 Blood collection and processing should be conducted as described in section 2.4, followed by the basic aggregometry protocol and the LTA assay (section 2.5.1).
2 Several PRP suspensions should be prepared in aggregometer cuvettes, and the aggregation curve of maximum-reversible platelet aggregation of hPRP induced by an agonist (PAF or thrombin) must be determined and characterised as 100% aggregation of platelets (Fig. 1B_1_).
3 Several concentrations of agonist (within a specific range) should be added to the aggregometer cuvette, which can induce reversible platelet aggregation in a concentration dependent manner (Fig. 1C). The higher the concentration of the agonist the higher the platelet aggregation curve detected by the aggregometer (Fig. 1C).
4 A linear relationship exists between the concentrations of an agonist (*i.e.* PAF or thrombin) within a specific range that induces platelet aggregation within the 20%–80% of the maximum-reversible platelet aggregation of hPRP. From this linear curve the concentration of the agonist require to induce 50% of platelet aggregation can be calculated, which is termed the EC_50_ value (half maximal effective concentration) of the specific agonist for platelet aggregation (Fig. 1D). The lower the EC_50_ value for an agonist the higher the potency of its platelet aggregation effect.

#### LTA for the evaluation of the inhibitory effects (IC_50_ values) of an antiplatelet agent against platelet aggregation of PRP

The application of LTA for evaluating the inhibitory effects of an antiplatelet agent either of natural, dietary or pharmacological origin against platelet aggregation of PRP induced by PAF or thrombin can be achieved as previously described [[12], [13], [14], [15], [16], [17], [18],^21^,^22^], with some modifications as previously described [1]. Briefly:

1 Blood collection and processing should be conducted as described section in 2.4, followed by the basic aggregometry protocol (Section 2.5.1) and the LTA assay (Section 2.5.2).
2 The aggregation curve of the maximum-reversible platelet aggregation of PRP (100% aggregation of platelets) induced by a specific concentration of an agonist (PAF or thrombin) in the absence of any antiplatelet agent is determined as the 0% inhibition (baseline).
3 Platelet suspensions of PRP are incubated in the presence of several concentrations of the antiplatelet compound for 2 min with stirring prior to the addition of any agonist.
4 By the addition of the same concentration of the agonist that was used for the baseline aggregation curve (100% of aggregation - 0% of inhibition) to the preincubated PRP cuvettes containing antiplatelet agent PRP suspensions, a reduction of the platelet aggregation curve should be observed (inhibitory effect) in relation to the baseline aggregation curve in a concentration dependent manner.
5 Generally, the higher the concentration of the antiplatelet agent in the aggregometer cuvette, the lower the platelet aggregation curve recorded by the aggregometer detector of the LTA assay, indicating the inhibitory effect of the antiplatelet agent on platelet aggregation as a percentage of inhibition in relation to the baseline aggregation curve (0% inhibition – 100% aggregation).
6 A linear relationship exists between the concentrations of the antiplatelet agent within a specific range that inhibits platelet aggregation within the 20%–80% of the maximum-reversible platelet aggregation of hPRP. From this linear curve the concentration of the agonist needed to achieve 50% inhibition of platelet aggregation can be calculated, which is known as the half maximal inhibitory concentration (IC_50_) value for the specific antiplatelet agent against platelet aggregation. The IC_50_ values reflect the inhibitory strength of each antiplatelet agent. The lower the IC_50_ value for an antiplatelet agent the higher its inhibitory effect against platelet aggregation induced by an agonist such as PAF or thrombin.

**Notes on assessing the inhibitory effects of potential antiaggregatory compounds:**

• The application of LTA for evaluating the inhibitory effects (IC_50_ values) of an antiplatelet agent against platelet aggregation of PRP induced by PAF or thrombin is important, since it provides information concerning the pathway that this antiplatelet compound can affect or inhibit (either that of the PAF/PAF-receptor pathway or that of the Thrombin/PAR-1 pathway, or both), and at which concentration these effects are induced [1,[12], [13], [14],[16], [17], [18], [19]]. These *in vitro* results are crucial for determining further research, either in cells and cell-related responses *in vitro* or *ex vivo/in vivo* in humans/animal-models.
• Suitable controls can be used before conducting the *in vitro* studies by using a known antagonist of the platelet agonist. For instance if testing with PAF and/or PAF-like molecules with agonistic effect [2], pharmacological inhibitors such as Rupatadine or CV-3988 may be used, or inhibitors of natural origin such as Ginkgolide B (*i.e.* BN52021) to assess and compare to the antiplatelet agent being tested as previously described [23,24].

## Conclusions

The methods and protocols described in this article can be applied as a tool for research in a basic and clinical setting by clinical researchers, biochemists, food and nutrition scientists and related laboratories who wish to assess platelet function and the effects of antiplatelet bioactive compounds on platelet aggregation induced by potent agonists such as PAF or thrombin using LTA. Relevant applications of the LTA assay and related methods and protocols described in this manuscript, can provide information about the health benefits or side-effects of various compounds on platelet function and cardiovascular health.

## Ethical statement

All experiments involving human subjects in the conceiving of these methods received ethical approval from The University of Limerick Ethics Committee and the protocols were devised and performed in accordance with the Declaration of Helsinki. All donors provided informed written consent.
